# Availability, Costs and Stock-Outs of Essential NCD Drugs in Three Rural Rwandan Districts

**DOI:** 10.5334/aogh.2729

**Published:** 2020-09-25

**Authors:** Jean Paul Mukundiyukuri, Jean Jacques Irakiza, Naome Nyirahabimana, Loise Ng’ang’a, Paul H. Park, Gedeon Ngoga, Ziad El-Khatib, Louis Nditunze, Etienne Dusengeyezu, Christian Rusangwa, Tharcisse Mpunga, Joel Mubiligi, Bethany Hedt-Gauthier

**Affiliations:** 1 Partners In Health/Inshuti Mu Buzima, Kigali, RW; 2 Ministry of Health, Kigali, RW; 3 Brigham and Women’s Hospital, Boston, US; 4 Department of Global Health and Social Medicine, Harvard Medical School, Boston, US; 5 Department of Public Health Sciences, Karolinska Institutet, Stockholm, SE

## Abstract

**Background:**

To reduce the non-communicable diseases (NCDs) burden, the World Health Organization has set a target to reach 80% availability of the affordable essential medicines required to treat NCDs by 2025.

**Objectives:**

This study described the availability, costs, and stock-outs of essential NCD drugs in three rural Rwandan districts.

**Methods:**

We retrospectively assessed 54 NCD drugs listed for district hospitals or health centers in the Rwanda national essential medicines list. Data were collected from three district hospitals and 17 health centers that host NCD clinics. We extracted data on drug availability, quantity dispensed, costs, stock-outs, and the replenishing supplier for these drugs between January 1 and December 31, 2017.

**Results:**

Overall, 71% of essential medicines for health centers and 78% of essential medicines for district hospitals were available at facilities. Only 15% of health centers experienced a stock-out of beclomethasone, while 77% experienced at least one stock-out of amlodipine and metformin. The median length of stock-out ranged from nine to 72 days, and 78% of the stock-outs across all health centers were replenished by a Non-Governmental Organization (NGO) partner. Except for enoxaparin and metformin, all district hospitals experienced at least one stock-out of each drug. The median length of stock-out ranged from 3.5 to 228 days, and 82% of the stock-outs across all district hospitals were replenished by the Rwandan Ministry of Health (RMOH). The least expensive drug was digoxin ($0.02, Interquartile range (IQR): 0.01, 0.10), while the most expensive was beclomethasone ($9.35, IQR: 3.00, 13.20).

**Conclusions:**

This study shows the viability of drug-supported NCD care in rural settings of sub-Saharan Africa. Stock-outs are a challenge; our study emphasizes the importance of the MOH/NGO partnerships in this context. Medicine costs are also challenging, though, in these districts, drugs are more affordable through community-based health insurance, government, and NGO partner subsidies.

## Introduction

Non-communicable diseases (NCDs), including cardiovascular, diabetes, and chronic respiratory diseases, are an increasing global health burden. Over 70% of deaths globally are attributed to NCDs, the majority of which occur in low- and middle-income countries (LMICs) [1,2]. In 2016, NCDs accounted for about 46% premature mortality among the population aged less than 70 years [3]. Within the next decade, Africa is projected to experience the largest relative increase in the population living with NCDs, with NCDs taking over as the leading cause of death [4] Without adequate intervention, the increasing NCD burden will cause additional constraints to health systems that are notably already overstretched and fragile [5]. This fragility is even more pronounced in rural areas where poor case detection, access to care, and documentation of NCDs has led to frequent under-estimation and under-prioritization of the endemic burden of NCDs.

Despite the increasing NCD burden, most people in LMICs have poor availability and access to the NCD medicines that are crucial for prevention and treatment [6,7]. In addition, existing evidence indicates that access and availability are disproportionate among the rural population compared to the urban population [8] and public facilities compared to private facilities [9]. Thus, the majority of patients residing in rural Africa and relying on public facilities for health care remain untreated or receive delayed NCD treatment.

In efforts to avert the growing NCD burden, the World Health Assembly endorsed the global action plan 2013–2020, which calls for a 25% relative reduction of NCDs related mortality by 2025. The nine global targets set to achieve this goal include reaching 80% availability of affordable essential NCD medicines, in both public and private facilities [10]. However, there is limited data on availability, stock-out, and cost of these medicines in rural sub-Saharan Africa (SSA).

In Rwanda, NCDs are rapidly becoming a major public health concern. In 2013, over 50% of all district hospitals outpatient clinic visits were attributed to NCDs [11], and in 2016, 45% of all deaths reported in Rwanda were attributed to NCDs [1]. Notably, 84% of the population in Rwanda resides in rural areas [12], where health care access and delivery is associated with a multitude of challenges [13]. To address this, in 2007, the Rwandan Ministry of Health (RMOH), in collaboration with Partners In Health/Inshuti Mu Buzima (PIH/IMB), began a novel NCD program to make care more accessible and affordable in three rural districts. In this study, we describe the availability of essential NCD drugs at the health facilities in these districts, the cost of these drugs to health facilities, and stock-outs levels of the drugs during 2017.

## Methods

### Study Setting and Intervention

This study was conducted in three rural Rwandan district hospitals – Butaro, located in the Northern Province and Kirehe and Rwinkwavu, located in the Eastern Province – and the 17 out of 28 surrounding health centers that have NCD programs supported by PIH/IMB. The three district hospitals are under the supervision of the RMOH and receive regular support from PIH/IMB, a non-governmental organization supporting the healthcare services in these three districts since 2005. In Rwanda, NCD care is decentralized to health centers where trained nurses use standardized treatment protocol to provide follow-up care for diagnosed NCD patients [14].

Nine of the 17 health centers host NCD clinics 2–3 times a week, and the remaining eight once per week. These clinics serve stable and mild to moderate NCD cases (asthma, hypertension, and non-insulin-dependent Type II diabetes), except for type 1 diabetes mellitus (managed at hospital level only) and are run by trained NCD nurses who also receive monthly supervision and mentorship from the NCD district hospitals nurses. Butaro, Kirehe, and Rwinkwavu District Hospitals host NCD clinics from Monday to Friday every week and serve patients with severe cases of NCDs or patients coming from catchment areas where the assigned health center does not have an NCD clinic. The hospital clinics are run by trained NCD nurses under the supervision of a medical officer. At both the health centers and hospitals, the clinical staff prescribes drugs as per the NCD protocols [15]. The patients who require admissions are followed in a hospital internal medicine service where they are cared for by internists.

Each health center and the hospital have an on-site pharmacy, which is under the control of the health facility’s administration. The district pharmacy, managed by the district pharmacist under local district administration, supplies medicines to both the district hospitals and health centers in its catchment area. While district hospital pharmacies are headed by pharmacists, health center pharmacies are headed by nurse store managers. Each pharmacy has two units – the main stock where the products from the district pharmacy or other sources are stored, and the dispensing point where patients receive medicines. Store managers are responsible for the distribution of drugs to the main stock and dispensing point. Each month, all pharmacies conduct physical inventory for each stocked product for further reporting and requisitioning to the district pharmacy. Accordingly, through monthly active distribution, the district pharmacy dispatches and delivers to each health facility depending on requested products and available stock.

When a district hospital or a health center makes a request to the district pharmacy, and the requested drugs are unavailable, the district pharmacy issues a signed product non-availability form allowing the hospital or health center to search for other possible sources, such as private wholesalers or partner organizations such as PIH/IMB. For each requisition, the health facility pharmacy pays in cash or receives an order on loan, payable within thirty days. PIH/IMB donates medicines free of charge. The cost to the patient is 120% of the cost to the facility; this is the mark-up margin recommended by the Rwanda Ministry of Health (RMOH) across all public health facilities in the country [16].

### Study Design and Population

In this retrospective study, we reviewed the availability, costs, and stock-outs (occurrence, length, and replenishment) of 54 Rwandan essential NCD drugs between January 1, 2017, through December 31, 2017, at Butaro, Kirehe, and Rwinkwavu District Hospitals and their surrounding health centers. We focused on three main groups of NCDs – cardiovascular diseases, diabetes, and chronic respiratory diseases. We included all drugs for these conditions that were on the Rwanda national essential medicines list (6^th^ edition of 2015) for treatment at district hospitals or health centers [17]. For cardiovascular diseases, this included 38 medicines for district hospital and 11 for health centers (Table 1); for diabetes, this list included seven medicines for district hospital and six for health centers; and for chronic respiratory diseases, the list included five medicines for district hospitals and four for health centers (Table 2).

**Table 1:** Drugs recommended for health facilities for cardiovascular disease care.

Therapeutic class	Drug name	Essential drug for HCs	Available in any of the study HCs in 2017?	Essential drug for DHs in 2017	Available in any of the study DHs in 2017?

ACE inhibitors	Captopril 12.5 mg, 25 mg tablets	Yes	Yes, *12.5 mg	Yes	Yes, *12.5 mg
	Lisinopril 5 mg tablets	No		Yes	Yes
Alpha-blockers	Methyldopa 250 mg	Yes	Yes	Yes	Yes
Angiotensin II receptor antagonist	Losartan 50 mg tablets	No		Yes	Yes
Antiplatelet	Acetylsalicylic Acid 100 mg tablets	Yes	Yes	Yes	Yes
Anticoagulants	Enoxaparin 40 mg/0.4 ml injection	No	_	Yes	Yes
	Heparin 5,000; 10,000 units/ml injection	No	_	Yes	Yes, *10,000 units/ml
	Warfarin 1 mg, 5 mg tablets	No	**_**	Yes	Yes
	Clopidogrel 75 mg, 300 mg tablets	No	**_**	Yes	No
Beta blockers	Atenolol 25 mg, 50 mg, 100 mg tablets	No	_	Yes	Yes, *25 mg
	Carvedilol 6.25 mg, 12.5 mg, 25 mg tablets	No	_	Yes	Yes
Calcium channel blockers	Amlodipine 5 mg, 10 mg tablets	Yes	Yes	Yes	Yes
	Nifedipine 10 mg, 20 mg tablets	No	**_**	Yes	Yes
Cardiac glycosides	Digoxin 250 μg/ml injection; 50 μg/ml oral liquid; 250 μg tablets	No	_	Yes	Yes, *50 μg/ml
Diuretics	Furosemide 20 mg, 40 mg; 10 mg/ml injection	Yes	Yes, *10 mg/ml	Yes	Yes, *20 mg
	Hydrochlorothiazide 25 mg tablets	Yes	Yes	Yes	Yes
	Mannitol 10%, 20% injection solution	No	_	Yes	Yes
	Spironolactone 25 mg tablets	Yes	Yes	Yes	Yes
Nitrate	Isosorbide dinitrate 5 mg, 20 mg sublingual tablets	No	_	Yes	Yes, *20 mg
Sympathomimetic	Dopamine 40 mg/ml injection	No	_	Yes	Yes
	Adrenaline 100 mg/ml (0.1%) injection	No	_	Yes	No
Vasodilator	Hydralazine 20 mg/ml injection	No	_	Yes	Yes

* Refers to the formulation/strength of the drug that was unavailable in the facility; HCs: Health centers; DHs: District hospitals.

**Table 2:** Drugs recommended for health facilities for diabetes and asthma care.

Therapeutic group	Drug name	Essential drug for HCs?	Available in any of the study HCs in 2017?	Essential drug for DHs?	Available in any of the study DHs in 2017?

Diabetes medicines	Glibenclamide 5 mg tablets	Yes	Yes	Yes	Yes
	Metformin 500 mg, 800 mg tablets	Yes	Yes, *800 mg	Yes	Yes, *800 mg
	Long-acting insulins 100 IU/ml injection	Yes	No	Yes	Yes
	Intermediate-acting insulins 30/70, 100 IU/ml injection	Yes	No	Yes	Yes
	Rapid-acting insulins 100 IU/ml injection	Yes	No	Yes	Yes
	Gliclazide 30 mg modified-release tablets	No	–	Yes	No
Asthma medicines	Aminophylline 250 mg/10 ml injection	Yes	Yes	Yes	Yes
	Beclomethasone inhaler 50 µg, 250 µg/metered dose	Yes	Yes	Yes	Yes
	Salbutamol inhaler 100 mcg/metered dose	Yes	Yes	Yes	Yes
	Salbutamol 5 mg/ml nebulizer solution	No	–	Yes	Yes

* Refers to the formulation/strength of the drug that was unavailable in the facility.

### Data Collection and Analysis

For each drug of interest, we extracted information from pharmacy stock-cards on drug formulation and strength, monthly consumption, drug cost, frequency of stock-outs, stock-out date, replenishment date, and the supplier who replenished the stock. Trained data collectors visited each health facility to retrieve this information onto paper data collection forms. The data collectors then transferred the data into an Excel database.

During analysis, we defined availability as to when medicine had a positive stock-balance verified using the stock-cards at the health facility at any time within the study period and calculated the percentage of facilities with drugs available during the study period. We calculated monthly consumption as median units of drugs dispensed per month. The cost of the drug, defined as the cost to the health facility, was also the median cost documented by the facilities during 2017. We collected the costs in Rwandan Francs and converted to US dollars ($) at the exchange of 850 Rwandan Francs to one United States Dollar. For tablets, we reported these costs for 100 tablets. We defined stock-out as when medicine recorded a zero-stock-balance at any point within the study period. We calculated the average number of stock-outs per facility by summing up the total number of stock-outs across all facilities and dividing them by the count of facilities with any stock-out of a given drug. For details on stock-outs, we restricted reporting only to the facilities that had the drug at any point in 2017, as indicated in Table 1 and Table 2. We analyzed data using Stata/IC version 15.1 (College Station, TX: StataCorp LLC) and present categorical data as frequencies and percentages and continuous data as average, standard deviations for these averages or medians and interquartile ranges (IQRs).

### Ethics

We received study approval from IMB Research Committee and ethics approval from the Rwanda National Ethics Committee (Kigali, Rwanda No. 102/RNEC/2018) and Partners Institutional Review Board (Boston, USA 2013P000047) to carry out this study. The pharmacists and head administrators approved data collection in their facilities.

## Results

### Health Centers

Across the 17 included health centers, there were 1,121 (56.8%) cardiovascular disease patients, 202 (10.2%) diabetic patients, and 649 (32.9%) asthma patients in care as of January 1, 2017 (Table 3). Of the 21 drugs that were on the Rwanda national essential medicines list for health centers, 15 (71.4%) drugs were ever available at any of the 17 health centers in 2017, including nine (81.8%) cardiovascular drugs, two (33.3%) diabetes drugs and four (100.0%) asthma drugs.

**Table 3:** Non-communicable disease patients active in study facilities as of January 2017.

Non-communicable disease Type	Patients in health centers n (%)	Drugs from the essential list available in the health center n (%)	Patients in district hospitals n (%)	Drugs from the essential list available in district hospitals n (%)

Cardiovascular	1121 (56.8)	9 (81.8)	880 (64.0)	29 (76.3)
Heart failure	9 (0.8)		393 (44.7)	
Hypertension	1112 (99.2)		487 (55.3)	
Diabetes	202 (10.2)	2 (33.3)	338 (24.6)	5 (71.4)
Type 1	–		67 (19.8)	
Type 2	131 (64.9)		240 (71.0)	
Missing	71 (35.1)		31 (9.2)	
Asthma	649 (32.9)	4 (100.0)	156 (11.4)	5 (100.0)
**Total**	**1972 (58.9)**	**15 (71.4)**	**1374 (41.1)**	**39 (78.0)**

#### Cardiovascular Drugs

Of the nine cardiovascular drugs available, only four drugs (44.4%) (captopril 25 mg tablets, acetylsalicylic acid 100 mg tablets, amlodipine 5 mg tablets, and hydrochlorothiazide 25 mg tablets) had at least 80% availability across health centers, and two (22.2%) (furosemide 20 mg tablets, spironolactone 25 mg tablets) were available at fewer than half of the health centers during 2017 (Table 4). Only two drugs (22.2%), namely captopril 25 mg tablets and hydrochlorothiazide 25 mg tablets, were available at all 17 health centers at some point in 2017.

**Table 4:** Availability, consumption, costs, stock-out trends, and replacement of essential cardiovascular drugs at health centers.

Therapeutic class	Type of drugs	HCs with drug available (n, %) (N = 17)	Median monthly consumption (IQR)	Median cost (US $) to the health facility (IQR)*	HCs with any stock-out (n, %)	Stock-outs per facility (n)	Average of stock-outs across facilities n (SD)	Median length of stock-out in days (IQR)	Stock-outs replaced by PIH (n, %)	Stock-outs replaced by MOH (n, %)

ACE inhibitors	Captopril 25 mg tablets	17 (100.0)	700 (191, 1495)	3.12 (2.56, 3.12)	12 (70.6)	33	2.8 (2.0)	17 (9, 27)	26 (78.8)	7 (21.2)
Alpha-blockers	Methyldopa 250 mg tablets	12 (70.6)	200 (0, 1000)	2.50 (2.50, 3.12)	8 (66.7)	25	3.1 (2.6)	18 (14, 34)	24 (95.8)	1 (4.2)
Antiplatelets	Acetylsalicylic acid 100 mg tablets^†^	14 (82.4)	0 (0, 1000)	0.45 (0.44, 0.54)	4 (28.6)	6	1.5 (0.5)	43 (23, 82)	0 (0.0)	5 (83.3)
Calcium channel blockers	Amlodipine 5 mg tablets	15 (88.2)	106 (0, 700)	4.20 (4.20, 6.97)	13 (86.7)	29	2.2 (1.0)	33 (12, 81)	22 (75.9)	7 (20.7)
	Amlodipine 10 mg tablets	12 (70.6)	210 (0, 1000)	3.21 (3.21, 11.06)	8 (66.7)	17	2.1 (1.1)	27 (9, 87)	12 (70.6)	5 (29.4)
Diuretics	Furosemide 20 mg tablets	2 (11.8)	0 (0, 45)	0.41 (0.41, 0.49)	0 (0.0)	0	N/A	N/A	N/A	N/A
	Furosemide 40 mg tablets^††^	9 (52.9)	0 (0, 1000)	0.49 (0.42, 1.00)	6 (66.7)	17	2.8 (2.1)	34 (9, 70)	14 (82.3)	1 (5.7)
	Hydrochlorothiazide 25 mg tablets^†^	17 (100.0)	728 (0, 1091)	1.18 (0.53, 3.00)	12 (70.6)	43	3.6 (1.8)	23 (14, 45)	36 (83.7)	6 (14.0)
	Spironolactone 25 mg tablets^†^	3 (17.6)	0 (0, 15)	5.20 (5.10, 5.70)	1 (33.3)	1	1.0 (0.0)	360 (360, 360)	0 (0.0)	0 (0.0)

* For tablets, we reported the cost for 100 tablets. ** One of the stock-outs was replaced from another source/supplier different from PIH or MOH. ^†^ Missing information on restock-details (supplier and restock-date) for one of the observed stock-outs in the facilities. ^††^ Missing information on restock-details (supplier and restock-date) for two of the observed stock-outs in the facilities. HCs: Health centers; IQR: Interquartile range; US $: United States Dollars; SD: Standard Deviation; PIH: Partners In Health; MOH: Ministry of Health; N/A: Not applicable.

Cardiovascular drugs with the highest median monthly unit dispensed at health centers were captopril 25 mg tablets (median = 700 tablets per month, IQR: 191, 1495), and hydrochlorothiazide 25 mg tablets (median = 728 tablets per month, IQR: 0, 1091). The following drugs had a median of zero units per month distributed: acetylsalicylic acid 100 mg tablets, furosemide 20 mg tablets, furosemide 40 mg tablets, and spironolactone 25 mg tablets. The least expensive cardiovascular drugs were acetylsalicylic acid 100 mg tablets ($0.45, IQR: 0.44, 0.54), furosemide 20 mg tablets (US$ 0.41, IQR: 0.41, 0.49) and furosemide 40 mg tablets ($0.49, IQR: 0.42, 1.00). The most expensive drugs for cardiovascular conditions were spironolactone 25 mg tablets ($5.20, IQR: 5.10, 5.70) and amlodipine 5 mg tablets ($4.20, IQR: 4.20, 6.97).

The percent of health centers that had a drug ever available that experienced any stock-out in 2017 ranged from 28.6% for acetylsalicylic acid 100 mg tablets to 86.7% for amlodipine 5 mg tablets (Table 4). The average number of stock-outs ranged from 1.0 (0.0) per year per facility for spironolactone 25 mg tablets to 3.6 (1.8) per year per facility for hydrochlorothiazide 25 mg tablets. The longest median stock-out length was for acetylsalicylic acid 100 mg tablets (43 days, IQR: 23, 82).

#### Diabetes and Asthma Drugs

Of the two diabetes and four asthma drugs, two (50.0%) asthma drugs were available at fewer than half of the health centers during 2017, namely: Aminophylline 250 mg/10 ml injection and beclomethasone inhaler 50 µg/metered dose (Table 5). Only one anti-diabetic drug, namely metformin 500 mg tablets, was available at all 17 health centers at some point in 2017.

**Table 5:** Availability, consumption, costs, stock-out trends, and replacement of essential diabetes and asthma drugs at health centers.

Type of drugs	HCs with drug available n (%) (N = 17)	Median monthly consumption (IQR)	Median cost (US $) to the health facility (IQR)*	HCs with any stock-out n (%)	Stock-outs per facility (n)	Average of stock-outs across facilities n (SD)	Median length of stock-out in days (IQR)	Stock-outs replaced by PIH (n, %)	Stock-outs replaced by MOH (n, %)

**Anti-diabetes**									
Glibenclamide 5 mg tablets^†^, **	15 (88.2)	124 (0, 1000)	0.43 (0.43, 0.6)	11 (73.3)	34	3.1 (1.9)	29.5 (13, 60)	30 (90.9)	2 (6.1)
Metformin 500 mg tablets^†^	17 (100.0)	800 (0, 1000)	1.18 (1.17, 1.65)	13 (76.5)	34	2.6 (1.6)	17.5 (9, 35)	26 (76.5)	7 (20.6)
**Anti-asthma**									
Aminophylline 250 mg/10 ml injection	1 (5.9)	0 (0, 1)	–	–	–	–	–	–	–
Beclomethasone inhaler 50 mcg/metered dose	2 (11.8)	2 (0, 6)	3.00 (3.00, 3.00)	0 (0.0)	0	0.0	N/A	N/A	N/A
Beclomethasone inhaler 250 mcg/metered dose	13 (76.5)	24 (10, 44)	3.00 (3.00, 3.00)	2 (15.4)	2	1.0 (0.0)	11 (1, 21)	2 (100)	0 (0.0)
Salbutamol inhaler 100 mcg/metered dose	16 (94.1)	14 (5, 35)	1.44 (1.44, 1.85)	11 (68.8)	30	2.7 (1.9)	9 (5, 22)	20 (66.7)	10 (33.3)

* For tablets, we reported the cost for 100 tablets. ** One of the stock-outs was replaced from another source/supplier different from PIH or MOH. ^†^ Missing information on restock-details (supplier and restock-date) for one of the observed stock-outs in the facilities. ^††^ Missing information on restock-details (supplier and restock-date) for two of the observed stock-outs in the facilities. HCs: Health centers; IQR: Interquartile range; US $: United States Dollars; SD: Standard Deviation; PIH: Partners In Health; MOH: Ministry of Health; N/A: Not applicable.

Metformin 500 mg tablets had the highest median monthly unit dispensed at health centers (median = 800 tablets per month, IQR: 0, 1000). The following anti-asthma drug had a median of zero units per month dispensed: Aminophyline 250mg/10 ml injection. Anti-diabetic glibenclamide 5 mg tablets ($ 0.43 IQR: 0.43, 0.60) were among the least expensive NCD drugs to the health centers. Among the most expensive NCD drugs were beclomethasone inhaler 50 µg/metered-dose and beclomethasone inhaler 250 µg/metered-dose, each costing US$3 (3.00–3.00).

The percent of health centers that had a drug ever available that experienced any stock-out in 2017 ranged from 15.4% for beclomethasone inhaler 250 mcg/metered dose to 76.3% for metformin 500 mg tablets (Table 5). The average number of stock-outs ranged from 1.0 (0.0) per year per facility for beclomethasone inhaler 250 mcg/metered dose to 3.1 (1.9) per year per facility for glibenclamide 5 mg tablets. The shortest median stock-out length was for salbutamol inhaler 100 mcg (nine days, IQR: 5, 22). For the 271 observed stock-outs across all NCD drugs at health centers, 212 (78.2%) were replaced by PIH/IMB.

### District Hospitals

Across the three district hospitals included in this study, there were 880 (64.0%) cardiovascular disease patients, 338 (24.6%) diabetic patients, and 156 (11.4%) asthma patients in care as of January 1, 2017 (Table 3). Of the 50 drugs that were on the Rwanda national essential medicines list for hospitals, 39 (78.0%) drugs were ever available at any of the three hospitals in 2017, namely 29 (76.3%) cardiovascular drugs, five (71.4%) diabetes drugs and five (100.0%) asthma drugs.

#### Cardiovascular Drugs

The drugs with the highest median monthly unit dispensed at the hospital were captopril 25 mg tablets (median = 13000 tablets per month, IQR: 4750, 16000); furosemide 40 mg tablets (median = 6000 tablets per month, IQR: 5000,9000), and nifedipine 20 mg tablets (median = 5950 tablets per month, IQR: 2140, 13050) (Table 6). The following drugs had a median of zero units per month dispensed: ACE inhibitor lisinopril 5 mg tablets, angiotensin II receptor antagonist losartan 50 mg tablets, anticoagulants enoxaparin 40 mg/0.4ml injection and heparin 5000 units/ml injection, beta-blocker carvedilol 25 mg tablets, calcium channel blocker nifedipine 10 mg tablets, cardiac glycosidase digoxin 250 µg/ml injection and digoxin 250 µg tablets, diuretic mannitol 20% injection solution and sympathomimetic dopamine 40 mg/ml injection. The least expensive cardiovascular drugs to hospitals were digoxin 250 µg tablets ($0.02, IQR: 0.01–0.10) and furosemide 10 mg/ml injection ($0.11, IQR: 0.07, 0.14) and the most expensive were warfarin 5 mg tablets ($57.00, IQR: 53.71, 57.00), warfarin 1 mg tablets ($29.69, IQR: 28.35, 31.04), and carvedilol 6.25 mg tablets ($20.85, IQR: 1.43, 40.28).

**Table 6:** Availability, consumption, costs, stock-out trends, and replacement of essential cardiovascular drugs at district hospitals.

	Type of drugs	DHs with drug available (n) (N = 3)	Median monthly consumption (IQR)	Median cost (US $) to the health facility (IQR)*	DHs with any stock-out (n)	Stock-outs per facility (n)	Average of stock-outs across facilities n (SD)	Median length of stock-out in days (IQR)	Stock-outs replaced by PIH (n, %)	Stock-outs replaced by MOH (n, %)

ACE inhibitors	Captopril 25 mg tablets	3	13000 (4750, 16000)	2.40 (1.99, 2.54)	2	10	5.0 (3.0)	13 (7, 18)	0 (0.0)	10 (100.0)
	Lisinopril 5 mg tablets^†^	3	0 (0, 1500)	–	2	6	3.0 (1.)	30 (10, 52)	0 (0.0)	5 (83.3)
Alpha-blocker	Methyldopa 250 mg tablets	3	2000 (1000, 30000)	3.25 (3.11, 3.45)	3	5	1.7 (0.9)	22 (12, 22)	0 (0.0)	5 (100.0)
Angiotensin II receptor antagonist	Losartan 50 mg tablets	1	0 (0, 50)	12.6 (12.6, 12.6)	1	2	2.0 (0.0)	157 (28, 286)	0 (0.0)	2 (100.0)
Antiplatelet	Acetylsalicylic acid 100 mg tablets	3	1500 (1000, 2000)	0.45 (0.40, 0.61)	3	9	3.0 (0.0)	20 (8, 35)	0 (0.0)	9 (100.0)
Anticoagulants	Enoxaparin 40 mg/0.4ml injection	1	0 (0, 7)	2.95 (2.95, 2.95)	0	–	–	–	–	–
	Heparin 5,000 units/ml injection	3	0 (0, 10)	5.97 (5.60, 7.52)	2	2	1.0 (0.0)	228 (245, 325)	0 (0.0)	1(100.0)
	Warfarin 1 mg tablets	3	200 (0, 500)	29.69 (28.35, 31.04)	3	7	2.3 (1.2)	40 (23, 159)	2 (28.6)	5 (71.4)
	Warfarin 5 mg tablets	3	500 (300, 722)	57.00 (53.71, 57.00)	3	11	3.7 (1.9)	14 (7, 40)	3 (27.3)	8 (72.7)
Beta-blockers	Atenolol 50 mg tablets	2	100 (0, 700)	1.87 (1.80, 2.25)	2	2	1.0 (0.0)	76 (14, 138)	0 (0.0)	2 (100.0)
	Atenolol 100 mg tablets^†^	3	500 (0, 650)	2.57 (2.25, 3.85)	3	7	2.3 (1.9)	4 (2, 14)	0 (0.0)	6 (85.7)
	Carvedilol 6.25 mg tablets	3	500 (0, 1600)	20.85 (1.43, 40.28)	3	4	1.3 (0.5)	32 (8, 89)	0 (0.0)	4 (100.0)
	Carvedilol 12.5 mg tablets	3	200 (0, 400)	10.15 (10.15, 10.15)	2	6	3.0 (2.0)	60 (15, 132)	0 (0.0)	6 (100.0)
	Carvedilol 25 mg tablets	2	0 (0, 500)	6.06 (1.62, 10.5)	1	1	1.0 (0.0)	60 (60, 60)	0 (0.0)	1 (100.0)
Calcium channel blocker	Amlodipine 5 mg tablets	3	1000 (200, 1000)	4.20 (1.69, 8.90)	2	8	4.0 (2.0)	15 (6, 36)	0 (0.0)	8 (100.0)
	Amlodipine 10 mg tablets	1	2000 (1900, 2350)	11.06 (11.06, 11.06)	1	1	1.0 (0.0)	21 (21, 21)	0 (0.0)	1 (100.0)
	Nifedipine 10 mg tablets	2	0 (0, 250)	2.1 (1.8, 2.5)	1	5	5.0 (0.0)	37 (15, 41)	0 (0.0)	5 (100.0)
	Nifedipine 20 mg tablets	3	5950 (2140, 13050)	1.89 (1.58, 2.43)	3	8	2.6 (1.2)	12 (7, 28)	0 (0.0)	8 (100.0)
Cardiac glycosidase	Digoxin 250 μg/ml injection	2	0 (0, 2)	0.52 (0.33, 4.48)	1	1	1.0 (0.0)	156 (156, 156)	0 (0.0)	1 (100.0)
	Digoxin 250 μg tablets^†^	3	0 (0, 0)	0.02 (0.01, 0.10)	3	6	2.0 (0.0)	40 (21, 121)	0 (0.0)	5 (83.3)
Diuretics	Furosemide 40 mg tablets	3	6000 (5000, 9000)	0.46 (0.42, 0.49)	3	12	4.0 (2.2)	4 (2, 11)	3 (25.0)	9 (75.0)
	Furosemide 10mg/ml injection	2	675 (494, 1050)	0.11 (0.07, 0.14)	1	9	9.0 (0.0)	4 (2, 9)	1 (11.1)	8 (88.9)
	Hydrochlorothiazide 25 mg tablets	1	1500 (0, 4500)	1.17 (1.17, 1.17)	1	2	2.0 (0.0)	90 (19, 161)	0 (0.0)	2 (100.0)
	Mannitol 10% injection solution^†^	2	1 (0, 5)	2.62 (1.65, 6.26)	2	3	1.5 (0.5)	27 (9, 205)	0 (0.0)	2 (66.7)
	Mannitol 20% injection solution	1	0 (0, 5)	2.06 (2.06, 2.06)	1	3	3.0 (0.0)	80 (3, 157)	0 (0.0)	3 (100.0)
	Spironolactone 25 mg tablets	3	2000 (2000, 2985)	5.1 (5.0, 5.6)	3	9	3.0 (1.6)	27 (7, 41)	0 (0.0)	9 (100.0)
Nitrate	Isosorbide dinitrate 5 mg sublingual tablets	2	1080 (300, 1980)	3.75 (3.75, 3.75)	2	6	3.0 (0.0)	12 (9, 17)	3 (50.0)	3 (50.0)
Sympathomimetic	Dopamine 40 mg/ml injection	3	0 (0, 1.5)	2.65 (1.47, 2.70)	2	3	1.5 (0.5)	59 (45, 103)	2 (66.7)	1 (33.3)
Vasodilator	Hydralazine 20 mg/ml injection	3	20 (2.5, 30)	7.41 (4.73, 7.53)	2	6	3.0 (1.0)	15 (9, 44)	1 (16.7)	5 (83.3)

* For Tabs, we reported the cost for 100 tablets. * For tablets, we reported the cost for 100 tablets. ** One of the stock-outs was replaced from another source/supplier different from PIH or MOH. ^†^ Missing information on restock-details (supplier and restock-date) for one of the observed stock-outs in the facilities. ^††^ Missing information on restock-details (supplier and restock-date) for two of the observed stock-outs in the facilities. HCs: Health centers; IQR: Interquartile range; US $: United States Dollars; SD: Standard Deviation; PIH: Partners In Health; MOH: Ministry of Health; N/A: Not applicable.

All hospitals that had a drug experienced at least one stock-out in 2017 for the following ten drugs: methyldopa 250 mg tablets, acetylsalicylic acid 100 mg tablets, warfarin 1 mg, and 5 mg tablets, atenolol 100 mg tablets, carvedilol 6.25 mg tablets, nifedipine 20 mg tablets, digoxin 250 µg tablets, furosemide 40 mg tablets and spironolactone 25 mg tablets (Table 6). For facilities with any stock-outs, the average number of stock-outs ranged from 1.0 (0.0) per year per facility for heparin 5000 units/ml injection, atenolol 50 mg tablets, carvedilol 25 mg tablets, amlodipine 10 mg tablets, and digoxin 250 µg/ml injection to 9.0 (0.0) per year per facility for furosemide 10 mg/ml injection. The shortest stock-outs were for atenolol 100 mg tablets, furosemide 40 mg tablets and furosemide 10 mg/ml injection (median = four days, IQR: 2,14) and the longest stock-outs was for heparin 5000 units/ml injection (median = 228 days, IQR: 245, 325). The RMOH replenished the supply for 50% or more of almost all district hospital stock-outs except for dopamine 40mg/ml injection.

#### Diabetes and Asthma Drugs

The drug with the highest average monthly unit dispensed was metformin 500mg tablets (median = 5000 tablets per month, IQR: 4000, 5300) (Table 7). Only one anti-asthma drug had a median consumption of zero units per month, namely beclomethasone inhaler 50 µg/metered dose. Anti-asthma drugs were among the least expensive NCD drugs, namely aminophylline 250 mg/10ml injection ($0.23, IQR: 0.18, 0.24) and the most expensive drug, namely beclomethasone inhaler 250 µg/metered dose ($9.35 IQR: 3.00, 13.20).

**Table 7:** Availability, consumption, costs, stock-out trends, and replacement of essential diabetes and asthma drugs at district hospitals.

Type of drugs	DHs with drug available (n) (N = 3)	Median monthly consumption (IQR)	Median cost (US $) to the health facility (IQR)*	DHs with any stock-out (n)	Stock-outs per facility (n)	Average of stock-outs across facilities n (SD)	Median length of stock-out in days (IQR)	Stock-outs replaced by PIH (n, %)	Stock-outs replaced by MOH (n, %)

**Anti-diabetes**									
Glibenclamide 5 mg tablets	3	2500 (2000, 40000)	0.59 (0.42, 0.76)	3	8	2.7 (0.9)	7 (4, 11)	0 (0.0)	8 (100.0)
Intermediate-acting insulins 30/70, 100 IU/ml injection	2	22 (10, 41)	5.45 (4.94, 7.36)	2	9	4.5 (0.5)	10 (6, 15)	2 (22.2)	7 (77.8)
Long-acting insulins 100 IU/ml injection**	3	43 (30, 50)	4.90 (4.65, 5.47)	2	8	4.0 (1.0)	8 (2, 13)	3 (37.5)	4 (50.0)
Metformin 500 mg tablets	3	5000 (4000, 5300)	1.70 (1.61, 1.81)	0	–	–	–	–	–
Rapid-acting insulins 100 IU/ml injection**	3	30 (20, 43)	4.65 (4.62, 5.48)	2	2	1.0 (0.0)	27 (0, 54)	1 (50.0)	0 (0.0)
**Anti-asthma**									
Aminophylline 250 mg/10 ml injection	3	2.5 (0, 26.5)	0.23 (0.18, 0.24)	3	5	1.7 (0.5)	37 (13, 46)	0 (0.0)	5 (100.0)
Beclomethasone inhaler 50 mcg/metered dose^†^	1	0 (0, 31)	3.00 (3.00, 3.00)	1	1	1.0 (0.0)	61 (61, 61)	0 (0.0)	0 (0.0)
Beclomethasone inhaler 250 mcg/metered dose	3	20 (12, 33)	9.35 (3.00, 13.20)	1	2	2.0 (0.0)	19 (15, 23)	2 (100.0)	0 (0.0)
Salbutamol inhaler 100 mcg/metered dose	3	56 (39, 60)	2.56 (2.30, 2.58)	2	4	2.0 (1.0)	11 (7, 15)	1 (25.0)	3 (75.0)
Salbutamol 5 mg/ml nebulizer solution	1	25 (5, 40)	5.60 (5.60, 5.65)	1	3	3.0 (0.0)	26 (10, 35)	3 (100.0)	0 (0.0)

* For Tabs, we reported the cost for 100 tablets. * For tablets, we reported the cost for 100 tablets. ** One of the stock-outs was replaced from another source/supplier different from PIH or MOH. ^†^ Missing information on restock-details (supplier and restock-date) for one of the observed stock-outs in the facilities. ^††^ Missing information on restock-details (supplier and restock-date) for two of the observed stock-outs in the facilities. HCs: Health centers; IQR: Interquartile range; US $: United States Dollars; SD: Standard Deviation; PIH: Partners In Health; MOH: Ministry of Health; N/A: Not applicable.

All the three hospitals experienced at least one stock-out in 2017 for glibenclamide 5mg tablets and aminophylline 250 mg/10ml injection (Table 7). For facilities with any stock-outs, the average number of stock-outs ranged from 1.0 (0.0) per year per facility for rapid-acting insulin 100 IU/ml injection vial and beclomethasone inhaler 50 mcg/metered dose to 4.5 (0.5) per year per facility for Intermediate-acting insulins 30/70, 100 IU/ml injection vial. The shortest stock-outs were for anti-diabetic glibenclamide 5 mg tablets (median = 7 days, IQR: 4, 11), and the longest stock-outs were for beclomethasone inhaler 50 mcg/metered dose (median = 61 days, IQR: 61, 61). The RMOH replenished the supply for 50% or more of almost all district hospital stock-outs except for rapid-acting insulin 100 IU/ml injection, vial beclomethasone inhaler 250 mcg/metered-dose, and salbutamol 5 mg/ml nebulizer solution. For the 196 observed stock-outs across all NCD drugs and district hospitals, 161 (82.1%) were replenished by the RMOH.

## Discussion

In this study, we describe the availability, consumption, cost, and stock-out of essential NCD medicines in three rural districts in Rwanda. Of the essential NCD medicines recommended in the Rwanda national essential medicines list, 71.4% were available in the health centers while 78.0% were available in the district level hospitals. These levels are slightly below the WHO’s 80% availability target for essential NCD medicines by 2025 but are generally higher when compared to the 7.5–54.4% reported by other studies conducted in LMICs [6,8,9,18]. This higher availability of NCD drugs in our study setting can be attributed to the integration of NCD care into the primary health care facility level and accompaniment of a partner NGO [11,19].

Despite the overall higher availability, some essential NCD drugs were not at all available during the study period. For example, insulins that are recommended at the health center level in the essential medicine list were not available. However, the national guidelines for the management of NCDs recommend the administration of insulin at the hospital level [17], and health center nurses are not trained in insulin administration, which may explain the non-availability of insulins at health centers. Nevertheless, medicines like warfarin and insulins are critical to be made consistently available to reduce the risk of mortality in post-cardiac surgery and Type I diabetes mellitus (T1DM) patients, respectively, which is an all too frequent problem across LMICs. Further, the inconsistent supply of medicines may cause inappropriate prescription where patients may not access comprehensive care within one health facility and lead to poor treatment adherence among patients [20] or may encourage patients to seek alternative treatment options such as traditional medicines [21]. Strategies to ensure all essential NCD drugs availability, such as streamlining a well-coordinated national quantification procurement and distribution system as it was done for HIV/AIDS drugs; public-private partnership for optimizing the supply of NCDs drugs; and reimbursement by health insurers at all level of health facilities are recommended.

Although availability is generally high in our study settings, nearly all drugs, both at the health centers and district hospitals, recorded at least one stock-out during the study period, stock-out rates tended to be higher for drugs with higher median consumption. Studies have shown that medicines stock-outs are common in SSA [22,23], and can be attributed to several factors such as distance to the drug repositories (district pharmacy/warehouse), ineffective communication, inadequate financing, inefficient supply system, and poor quantification of medicines [22,24,25]. The challenges of stock-outs could be overcome by involving all actors in the health supply chain and putting in place strategies to address gaps in the system and engender the necessary options for reforms. Those strategies could include, among others, strengthening existing medicines supply chain management system through careful selection of cost-effective medicines, a transparent procurement system with the prequalification of reliable suppliers, accurate and timely reporting system on medicines consumption, adequate financing, performance monitoring, and compliance by both health system staff and suppliers [26,27,28,29].

Drug costs can make NCD treatment inaccessible to rural African patients. What we report in this paper are the costs of these drugs to the health facilities. In Rwanda, the costs to the patients in both public and private health facilities are set at a maximum of 20% margin over the facility cost, as instructed by the RMOH [16,30]. However, it is important to note that patients in these districts often do not pay the full price as 97% of Rwandans have Mutual Health Insurance [12], which typically covers 90% of the cost of drugs for patients. This type of insurance scheme is largely unavailable in most other SSA countries. Even with this subsidy, the insurance co-pay of 10% can be prohibitive to people from a low socio-economic background. To alleviate this financial burden and promote drug access, the government of Rwanda provides 100% medical coverage which is also not available in most other SSA countries, including prescription drugs, to the poorest identified through the national Ubudehe systems families who do not own a household and can hardly afford basic needs. Given the chronic nature of the NCDs, patients incur additional financial burden to acquire the lifelong treatment drugs. We, therefore, recommend that the government extends the 100% medical coverage to population in subsequent categories especially for vital drugs such as insulins. In addition, organizations like the World Health Organization (WHO), Ministries of Health and other partners should focus on strategies that enhance universal access to NCD medicines such as providing free insulin to T1DM patients who are dependent on exogenous insulin for normal metabolism.

The study findings suggest that the availability and stock-out patterns of the essential NCD medicines in Rwanda vary across health center and district hospitals. Though more patients were seen at health centers, the availability of medicines was higher at district hospitals than health centers. That might justify the reason why consumptions were noted greater at hospitals than health centers. Further, stock-outs were noted 1.4 times at health centers than hospitals (271 stock-outs across all health centers and 196 across hospitals). This might be related to the different supply channels used by those facilities levels, health conditions managed at each health facility level, management capabilities of pharmacy managers at the facility level.

There are several limitations that should be considered in interpreting our results. First, these facilities are supported by an NGO, and the results may not generalize to other settings of SSA. In addition, the use of secondary data collected from logistics tools (stock-cards and price lists) limited the type of information we could obtain for our study. Due to time constraints, we were unable to check whether no discrepancy existed between the actual stock on hand and the balance on the stock card. Also, we were unable to collect information on whether alternative formulations were used during stock-outs, the reasons for stock-outs, and the actual costs to patients. We also used any positive stock balance as a lack of stock-out, although this balance might have been low for some drugs. Although we did not compare the stock values in-stock cards with the physical stock, the pharmacy staff completes regular inventory audits to ensure the accuracy of the stock card information. This information, along with studies on the correct prescribing of NCD medicines and patient adherence to NCD drugs, needs further investigation.

## Conclusions

The availability, stock-out, and cost of medicines are still a challenge in providing NCD services in sub-Saharan Africa. The few studies to date have focused on urban or tertiary facilities. Our results show that NCD drugs can be available in rural and decentralized facilities, but with frequent stock-outs. Countries must intensify efforts and strengthen health supply chain management systems up to lower health facility levels. Partner organizations can help both strengthen systems and also supply medicines when the national supply chain has failed. Other programs can learn about making drugs affordable by studying the community-based insurance scheme in Rwanda that provides free drugs to the very poor and a fixed 10% co-payment to the rest of the population. WHO and other partners should provide more advocacy for less expensive NCD drugs – especially insulins – which should be free for Type 1 diabetes mellitus and promote the manufacturing of affordable generic insulins. We also recommend follow up studies on determinants of drug availability and affordability, the alignment of national treatment guidelines and essential medicines list, and modify insurance benefits designs to mitigate prescription drug cost burdens for diabetes Type 1 diabetes mellitus patients.

## References

[1] Organización Mundial de la Salud. IDF Diabetes Advocacy Toolkit – 2017 NCDs Progress Monitor. Geneva; 2017.

[2] Lozano R, Naghavi M. Global and regional mortality from 235 causes of death for 20 age groups in 1990 and 2010: A systematic analysis for the Global Burden of Disease Study. Elsevier; 2010. 10.1016/S0140-6736(12)61728-0 PMC10790329 23245604

[3] WHO-Global Health Observatory data. Premature NCD deaths. WHO. https://www.who.int/gho/ncd/mortality_morbidity/ncd_premature/en/. Accessed June 14, 2019.

[4] Marquez P, Bank W. The Challenge of Non-Communicable Diseases and Road Traffic Injuries in Sub-Saharan Africa: An Overview. 2013.

[5] Boozary AS, Farmer PE. The Ebola Outbreak, Fragile Health Systems, and Quality as a Cure. JAMA. 2014; 312(18): 1859 DOI: 10.1001/jama.2014.14387 25285459

[6] Mendis S, Fukino K, Cameron A, et al. The availability and affordability of selected essential medicines for chronic diseases in six low- and middle-income countries. Bull World Health Organ. 2007; 85: 279–288. DOI: 10.2471/BLT.06.033647 17546309 PMC2636320

[7] Chow CK, Ramasundarahettige C, Hu W, et al. Availability and affordability of essential medicines for diabetes across high-income, middle-income, and low-income countries: a prospective epidemiological study. Lancet Diabetes Endocrinol. 2018; 6(10): 798–808. DOI: 10.1016/S2213-8587(18)30233-X 30170949

[8] Khatib R, McKee M, Shannon H, et al. Availability and affordability of cardiovascular disease medicines and their effect on use in high-income, middle-income, and low-income countries: an analysis of the PURE study data. Lancet. 2016; 387(10013): 61–69. DOI: 10.1016/S0140-6736(15)00469-9 26498706

[9] Cameron A, Ewen M. Medicine prices, availability, and affordability in 36 developing and middle-income countries: A secondary analysis. Lancet. 2009; 373(9659): 240–249. DOI: 10.1016/S0140-6736(08)61762-6 19042012

[10] World Health Organisation. Global action plan for the prevention and control of non-communicable diseases 2013–2020. 2013.

[11] MOH R. Republic of Rwanda Non Communicables Disease Policy. Kigali; 2015.

[12] National Institute of Statistics of Rwanda (NISR). Rwanda Demographic and Health Survey, 2014–2015. 2016.

[13] Strasser R, Kam SM. Rural health care access and policy in developing countries. Annu Rev Public Health. 2016; 37(1): 395–412. DOI: 10.1146/annurev-publhealth-032315-021507 26735432

[14] Ndayisaba A, Harerimana E, Borg R, et al. A clinical mentorship and quality improvement program to support health center nurses manage type 2 diabetes in rural Rwanda. J Diabetes Res. 2017; 2017: 1–10. DOI: 10.1155/2017/2657820 PMC5738565 29362719

[15] Bukhman G, Kidder A. The PIH Guide to Chronic Care Integration for Endemic Non-Communicable Diseases. Boston, Massachusetts: Partners In Health; 2011.

[16] Seto MC. Performance Audit Report On Procurement And Management Of Drugs And Medical Supplies And Its Impact On Health Care At University Teaching Hospital- Kigali (UTH-K/CHUK). 2016; 28 DOI: 10.1177/1079063216639485

[17] MoH-Rwanda. National List of Essential Medicines for Paediatrics. Kigali; 2015.

[18] Ewen M, Zweekhorst M. Baseline assessment of WHO’s target for both availability and affordability of essential medicines to treat non-communicable diseases. Podobnik B (ed.), PLoS One. 2017; 12(2): 0171284 DOI: 10.1371/journal.pone.0171284 PMC5295694 28170413

[19] Bukhman G, Kidder A. PIH Guide to Chronic Care Integration for Endemic Non-Communicable Diseases: Rwanda Edition. Boston, Massachusetts: Patners in Health; 2011.

[20] Lulebo AM, Mutombo PB, Mapatano MA, et al. Predictors of non-adherence to antihypertensive medication in Kinshasa, Democratic Republic of Congo: A cross-sectional study. BMC Res Notes. 2015; 8(1): 526 DOI: 10.1186/s13104-015-1519-8 26427798 PMC4591704

[21] Mwaka AD, Okello ES. Barriers to biomedical care and use of traditional medicines for treatment of cervical cancer: An exploratory qualitative study in northern Uganda. Eur J Cancer Care (Engl). 2015; 24(4): 503–513. DOI: 10.1111/ecc.12211 24923866 PMC4930140

[22] Wagenaar BH, Gimbel S, Hoek R, et al. Stock-outs of essential health products in Mozambique – longitudinal analyses from 2011 to 2013. Trop Med Int Heal. 2014; 19(7): 791–801. DOI: 10.1111/tmi.12314 PMC4479203 24724617

[23] Onchweri Albert OB. Availability of Essential Medicines and Supplies during the Dual Pull-Push System of Drugs Acquisition in Kaliro District, Uganda. J Pharm Care Heal Syst. 2015; 2 DOI: 10.4172/2376-0419.S2-006

[24] Beran D, Ewen M. Availability and Affordability of Essential Medicines: Implications for Global Diabetes Treatment. Curr Diab Rep. 18(8): 48 DOI: 10.1007/s11892-018-1019-z 29907884

[25] Mori AT, Owenya J. Stock-outs of antiretroviral drugs and coping strategies used to prevent changes in treatment regimens in Kinondoni District, Tanzania: A cross-sectional study. J Pharm Policy Pract. 2014; 7(1): 3 DOI: 10.1186/2052-3211-7-3 25848543 PMC4366935

[26] Lim SS, Vos T, Flaxman AD, et al. A comparative risk assessment of burden of disease and injury attributable to 67 risk factors and risk factor clusters in 21 regions, 1990–2010: A systematic analysis for the Global Burden of Disease Study 2010. Lancet. 2012; 380(9859): 2224–2260. DOI: 10.1016/S0140-6736(12)61766-8 23245609 PMC4156511

[27] Medicine AOF. Improving Availability and Accessibility of Medicines: A Tool for Increasing Healthcare Coverage. Arch Med. 2015; 7(5): 1–9.

[28] Management Sciences for Health. MDS-3. Managing Access to Medicines and Health Technologies. Arlington, VA 2012.

[29] WHO. Essential Medicines and Basic Health Technologies for Non-communicable Diseases: Towards a Set of Actions to Improve Equitable Access in Member States. 2015.

[30] MOH-Rwanda. Amendment on the Ministerial Circular No 20/3425/PTF/2009 on the procurement and distribution of medicines and other medical supplies on the national territory. 2020.

